# Levels and Distributions of ^210^Pb and ^210^Po in Selected Seafood Samples in China and Assessment of Related Dose to Population

**DOI:** 10.3390/ijerph18063036

**Published:** 2021-03-16

**Authors:** Xiangyin Kong, Yuxin Qian, Qishan Zheng, Yanqin Ji

**Affiliations:** 1 China CDC Key Laboratory of Radiological Protection and Nuclear Emergency, Chinese Center for Disease Control and Prevention, National Institute for Radiological Protection, Beijing 100088, China; kongxiangyin@nirp.chinacdc.cn (X.K.); qianyuxin@nirp.chinacdc.cn (Y.Q.); 2 Fujian Center for Prevention and Control of Occupational Diseases and Chemical Poisoning, Fuzhou 350025, China; zqs_73@163.com

**Keywords:** ^210^Pb, ^210^Po, activity concentration, seafoods, dose, radiological risk

## Abstract

In this study, the activity concentrations levels of ^210^Pb and ^210^Po in the edible portions of eight seafood samples collected from the Fujian coast of China were determined. The activity concentrations ranged from 0.74 ± 0.08 to 12.6 ± 1.0 Bq/kg for ^210^Po and from the minimum detectable limit (MDL, 0.80 Bq/kg) to 11. 7 ± 1.1 Bq/kg for ^210^Pb. The ^210^Po activity concentration in all the fish organs ranged from 0.68 to 204 Bq/kg (*w.w.*), and the ^210^Po activity was mainly concentrated in the stomach, spleen, heart, liver, gonad, and intestine samples. The ^210^Pb activity concentration in all the fish organs ranged from the MDL to 15.2 Bq/kg (*w.w.*), and the ^210^Pb activity was concentrated in the head, fish scale, and gill samples. The annual effective ingestion doses ranged from 82.8 to 255 μSv/a for all age groups, and the lifetime risk of cancers were estimated. Both the effective ingestion doses and cancer risk to humans were within the acceptable ranges.

## 1. Introduction

Natural radionuclides ^210^Pb (T_1/2_ = 22.3 a) and ^210^Po (T_1/2_ = 138.4 d) are members of the ^238^U decay chain [1]. ^210^Pb and ^210^Po enter the marine environment from atmospheric deposition at the ocean surface, from the in situ radioactive decay of ^226^Ra dissolved in seawater, from the decay of ^222^Rn gas exhaled by the seafloor, and from river and anthropogenic discharges. Polonium ions in the marine environment are rapidly adsorbed onto suspended particles and are accumulated by marine organisms while lead ions are adsorbed onto inorganic particles; thus, ^210^Po can be accumulated in marine biotas more effectively than ^210^Pb [2]. Early research on ^210^Po and ^210^Pb levels and their distribution in marine organisms indicated that ingestion of seafood could be the main exposure pathway for humans to receive radiation [3]. According to the UNSCEAR (United Nations Scientific Committee on the Effects of Atomic Radiation) report, the annual effective doses caused by the ingestion of ^210^Pb and ^210^Po were approximately 80% of that caused by the ingestion of uranium- and thorium-series radionuclides [1]. Consequently, investigating the radiological impact of ^210^Po and ^210^Pb in marine products is necessary for protecting human health.

The ^210^Pb and ^210^Po activity concentrations in marine biota are spread over several orders of magnitude in the literature [4,5,6,7]. Previous studies focused on the activity concentrations of edible portions of seafood and the related dose assessment to the human [4,8,9,10,11,12,13,14,15,16]. The ^210^Pb and ^210^Po activity concentrations and ^210^Po/^210^Pb ratios in separate organs of different marine biota are essential for establishing reference individuals/reference marine biota for use in radiation protection [17], are important for understanding of the ^210^Po-enriched biochemistry and are critical for establishing the relevant standards for the limits/reference level of radionuclides in food samples.

In China, investigation of the ^210^Po and ^210^Pb activity in marine biota started relatively late, and few studies have been performed. In recent years, studies have focused on the edible portions of common marine biota [18,19,20]. However, the distributions of the ^210^Pb and ^210^Po activity concentrations in selected marine organisms have rarely been reported, and the current understanding of the ^210^Po-enriched biochemistry is poor. Thus, the main objectives of the present study were to determine the activity concentrations of ^210^Po and ^210^Pb in edible portions and all other organs of various marine biota species in China and to evaluate the annual effective ingestion dose and lifetime risk of cancer to the public in terms of health and safety.

## 2. Materials and Methods

### 2.1. Sample Collection and Preparation

**Figure 1:**
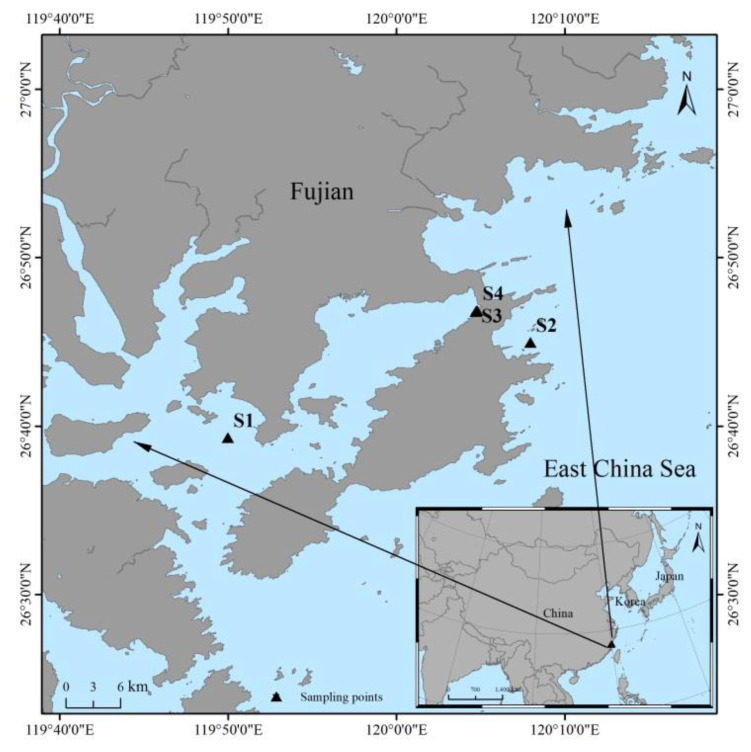
Locations of sampling sites on Fujian coast, China. S is sampling site.

Eight seafood samples, including fish, crustacean, and algae samples, were collected from the Fujian coast, China, in August 2020. The research area is shown in Figure 1. The sampling site, corresponding samples, and feeding habitat are presented in Table 1. All the seafood samples were classified, marked, and transfer to the laboratory on the same day. These samples were cleaned with ultrapure water to eliminate any possible residues and impurities. The edible portions (muscles) of the fish samples were extracted from every biota, and the non-edible portions of the fish were divided into 12 organs: the head, fish fins, bones, scales, gills, kidney, stomach, spleen, heart, liver, gonad, and intestine. The non-edible portions of the crustaceans were divided into the head and carapace. All the samples were labeled, weighed, and dried at 80 °C to a constant weight (to avoid ^210^Po losses) and finally crushed and homogenized for analysis.

### 2.2. Apparatus and Reagents

Liquid scintillation counting (Tri-Carb 3170TR/SL, PerkinElmer, Walsham, MA, USA) was used to measure the ^210^Pb and ^210^Po activity. The elemental concentrations of Pb were measured via inductively coupled plasma mass spectrometry (Element II, Thermo Fisher Scientific, Bremen, Germany). A microwave digestion system (Preeken EXCEl, Shanghai, China) was used for digestion of the seafood samples.

All the chemicals used were of analytical grade (AR, Beijing, China). Sr·spec resin (100–150 μm) was purchased from Triskem International (Rennes, France). A ^210^Pb reference solution (equilibrium with ^210^Bi and ^210^Po, 363.8 Bq/g) purchased from National Physical Laboratory (NPL, Tdington, London) was used for ^210^Pb and ^210^Po efficiency correction. A Pb carrier was prepared via the dissolution of Pb(NO_3_)_2_ (AR, Beijing, China) for Pb chemical recovery correction. Ultima Gold™ AB was purchased from PerkinElmer (USA). Ultrapure water (18.2 MΩ·cm^−1^) obtained from a Milli-Q system (Millipore, Billerica, MA, USA) was used.

### 2.3. Sample Digestion, Separation, and Measurement

The radiochemical procedure used for the ^210^Pb and ^210^Po activity concentrations in seafood samples is given elsewhere [21]. In brief, 0.5 g dried seafood samples were digested by the microwave digestion system. The ^210^Pb and ^210^Po in the samples were separated from the digestive liquid using a previously prepared Sr-Spec column (2 mL). 20 mL of 2 M HCl solution, 5 mL of 1 M HNO_3_ + 25 mL 0.1 M HNO_3_ and 20 mL 0.1 M (NH_4_)_2_C_2_O_4_ successively passed through the column for ^210^Bi (discard), ^210^Po and Pb fraction striping, respectively. The ^210^Pb eluents and ^210^Po eluents were heated gently to near dryness, and several small amounts of 0.1 M HNO_3_ were also added to homogeneity. 2 mL 0.1 M HNO_3_ and 18 mL of Gold AB liquid scintillation cocktail were added, shaken well, and placed in the dark for 2 h, the ^210^Pb and ^210^Po activity concentrations were measured via liquid scintillation counting (LSC) in the α/β pulse discrimination analysis (PSA) mode (PSA value = 145) for 1000 min, the Region of Interest (ROI) for ^210^Pb and ^210^Po were 1–28 channels and 0–1000 channels, respectively. Then, 2 mg stable Pb carrier was added in each samples before seafood samples digestion for Pb recovery correction, Pb recovery was calculated by initial concentration and final concentration after isolated by Sr-Spec column. The concentration of Pb was determined by inductively coupled plasma mass spectrometry (ICP-MS). The Po yield of this procedure was expressed as overall efficiency, which was determined by measuring the net counts of ^210^Po before and after separated on Sr·spec column. Batch experiments indicate that overall efficiency of Po were fluctuated in a small range (66.7 ± 2.5%), ^210^Po overall efficiency was recommended for calculation the activity concentrations of ^210^Po in further experiments.

### 2.4. Method Validation

To validate the method, a reference material (IAEA-447, Moss Soil reference material, International Atomic Energy Agency, Vienna, Austria) was used. The results obtained using our method agreed well with certified values. Additionally, to validate the results, the ^210^Po activity concentration of every sample was determined via α spectrometry after source preparation through spontaneous deposition onto a copper plate, which is the most commonly used method for monitoring the activity of ^210^Po [22].

### 2.5. Evaluation of Effective Ingestion Dose and Lifetime Risk of Cancer to Humans

The effective ingestion dose to humans from marine seafood ingestion was evaluated according to the seafood consumption per year, dose conversion factors for humans and activity concentrations of seafood. The formula for radionuclide estimation is [23,24]:

(1) $$C_{D}=R_{c}\times I_{R}\times D_{F}$$

where *C*_D_ represents the annual effective ingestion dose (Sv/a), *R*_C_ represents the activity concentration of the specific radionuclides in the seafood samples (Bq/kg), *I*_R_ represents the annual ingestion rate (kg/a), and *D*_F_ is the dose conversion factor (Sv/Bq).

The parameter used for annual intake rate (*I*_R_) in this study was adopted from a previously reported method [25]. *I*_R_ for adults (>18 years old), juveniles (14–17 years old) are 14.60 kg/a, and that for children (7–13 years old) is 10.95 kg/a. The parameters in Table 2 for the dose conversion factor (*D*_F_) were obtained from International Atomic Energy Agency (IAEA) [26].

The lifetime risk of cancer was also calculated via the formula [23,24]:

(2) $$R_{K}=I_{R}\times f_{1}\times T\times R_{coe}\times R_{c}$$

where *R*_K_ represents the lifetime risk of cancer, *I*_R_ represents the annual seafood ingestion rate (kg/a), *f*_1_ represents the estimated gastrointestinal absorption fraction of a specific radionuclide, *T* represents the exposure duration (50 a. for adults, 60 a. for juveniles, and 70 a. for children), *R*_coe_ is the risk coefficient from ICRP [27], and *R*_C_ represents the activity concentration of the specified radionuclide in the seafood samples (Bq/kg).

## 3. Results and Discussion

### 3.1. ^210^Po and ^210^Pb Concentrations in Edible Tissues of Different Biota Species

The activity concentrations of ^210^Po and ^210^Pb in the edible portions of seafood samples are presented in Table 3. All the data have units of Bq/kg wet weight (*w.w.*). The average ^210^Po activity concentration was 5.31 ± 0.52 Bq/kg for all the edible portions of the seafood samples and ranged from 0.74 ± 0.08 Bq/kg (Red Sea bream muscle samples) to 12.6 ± 1.0 Bq/kg (eel samples (*Anguillidae*)), while the ^210^Pb activity concentrations ranged from the minimum detectable limit (MDL), i.e., 0.8 Bq/kg wet weight for ^210^Pb) to 11.7 Bq/kg (eel samples), with a mean value of 3.36 ± 0.41 Bq/kg. The ^210^Po and ^210^Pb concentrations in seafood samples found in our study were compared to those determined in previous studies from China and other countries. Our results are consistent with the previously reported ranges of 0.13–3.26 Bq/kg (*w.w.*) and 0.2–25.8 Bq/kg (*w.w.*) for the ^210^Pb and ^210^Po activity concentrations, respectively, in seafood samples from the coast of Guangdong, China [20]. They are also similar to a previously reported finding that the ^210^Po level of various seafood edible portions ranged between 1.17 × 10^−1^ and 6.58 × 10 Bq/kg (*w.w.*) [18]. Additionally, the ^210^Po activity concentration in the seafood samples agreed well with the results of a seafood radionuclide survey conducted in 1977–1978 [28].

To estimate the ^210^Po and ^210^Pb activity concentrations of seafood samples from coastal regions of China, we concluded the previously reported activity concentrations of ^210^Po and ^210^Pb published by other countries. Compared with results from Spain, India, and Turkey [11,29,30], the concentrations of ^210^Po in our survey were lower, and there were no significant differences in the concentration range of ^210^Pb. The ^210^Po and ^210^Pb activity concentrations in our survey were similar to those reported for other countries [4,8,12,13,14,16,24,31,32,33,34,35]. Significant differences in the ^210^Po and ^210^Pb activity concentrations among different countries were observed, possibly owing to the marine species evaluated and the variations in the geochemistry of the regions [8]. The average activity concentration of ^210^Po in fish samples was slighter lower than the representative concentrations reported by UNSCEAR (2.4 Bq/kg) [1]. The average activity concentrations of ^210^Po in the crustacean samples were slightly higher than the representative concentrations reported by UNSCEAR (6.0 and 15 Bq/kg, respectively). The ^210^Po concentration decreased in the following order: crustaceans > fish > algae. The higher ^210^Po activity concentrations in the crustaceans are explained as follows: (1) the edible portions of the crustaceans contained the digestive system, and the ^210^Po activity concentrations were generally higher in the internal organs than in the muscles [17]; (2) the crustaceans were captured in nature and were not subjected to artificial feeding. The order of the ^210^Pb activity was identical to that for ^210^Po. The ^210^Po/^210^Pb ratio was >1 for almost all the samples, with the exception of the Carassius auratus auratus and Largehead hairtail (*Trichiurus lepturus*) samples. This may be because the ^210^Po was more easily concentrated in the internal organs than the ^210^Pb.

### 3.2. Distributions of ^210^Po and ^210^Pb Activity Concentrations in Selected Organs of Different Species

**Figure 2:**
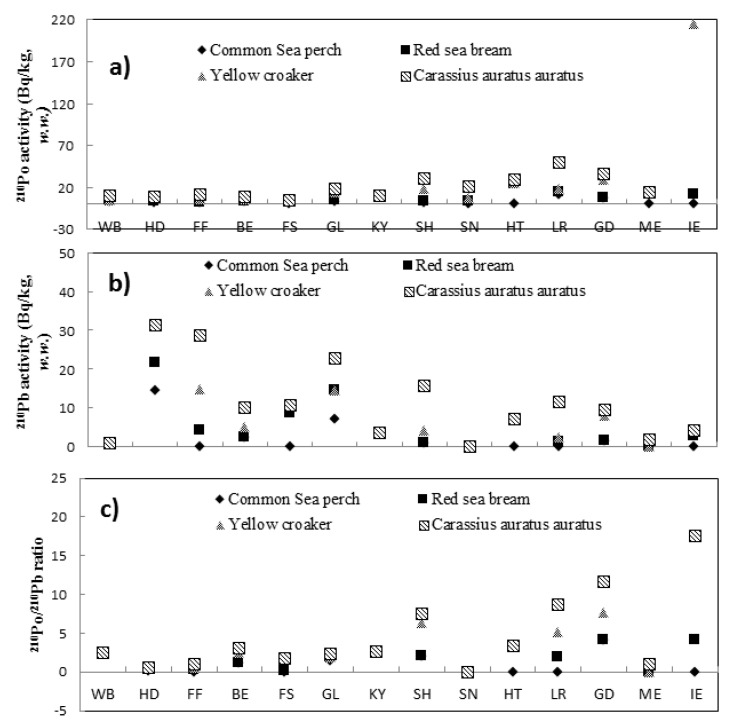
Distributions of the ^210^Po and ^210^Pb activity concentrations in selected organs of different species. (**a**) is for ^210^Po activity concentration; (**b**) is for ^210^Pb activity concentration; (**c**) is for ^210^Po/^210^Pb ratio). Note: WB = whole body, HD = head, FF = fish fin, BE = bone, FS = fish scale, GL = gill, KY = kidney, SH = stomach, SN = spleen, HT = heart, LR = liver, GD = gonad, ME = muscle, IE = intestine.

The ^210^Po and ^210^Pb activity concentrations and ^210^Po/^210^Pb ratios in selected organs of four types of fish are shown in Figure 2.

As shown in Figure 2a, The ^210^Po activity concentrations in all the fish organs ranged from 0.68 to 204 Bq/kg (*w.w.*). The lowest and highest values were obtained for the fish scale sample of the Red sea bream and the intestine sample of the yellow croaker, and relatively high values were obtained for the stomach, spleen, heart, liver, gonad, and intestine samples. These results are consistent with previous studies [2,3]. As shown in Figure 2b, the ^210^Pb activity concentrations in all the fish organs ranged from the MDL to 15.18 Bq/kg (*w.w.*), and the highest value was obtained for the head sample of the Common sea perch. Relatively high values were obtained for the head, fish scale, and gill samples. Regarding the ^210^Po/^210^Pb ratio, the values for the stomach, liver, gonad, and intestine samples were significantly higher than one, and those for the bone, gill, head, fish scale, and fish fin samples were generally lower than one. This may be because ^210^Pb and ^210^Po accumulated in the organisms through the food chain (less ^210^Po absorption in the form of inorganic ions but more organic ^210^Po). ^210^Pb was mainly deposited on bones, and ^210^Po was mainly deposited on internal organs such as the liver, gastrointestinal tract, and gonad [17]. Similarly, the ^210^Po activity concentration of muscle samples in crustaceans (shrimp and *Oratosquilla oratoria*) was significantly lower than that of head samples, which may contributed to the viscera and digestive system of these two species are inside the head samples, and the viscera and digestive system were strongly bonded to ^210^Po, while the ^210^Pb activity concentrations exhibited no significant differences.

### 3.3. Effective Ingestion Dose and Risk to Humans via Seafood Consumption

**Figure 3:**
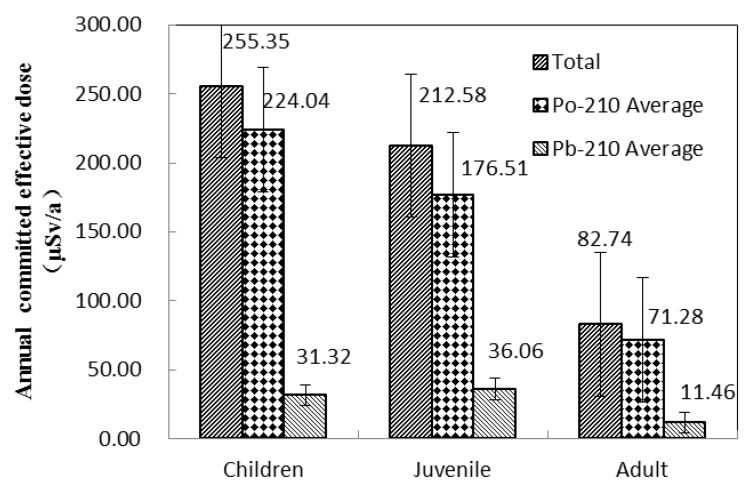
Effective ingestion doses due to the ingestion of ^210^Pb and ^210^Po through seafood consumption for different ages.

The effective ingestion doses due to the ingestion of ^210^Pb and ^210^Po through seafood consumption for different ages were estimated, as shown in Figure 3. Because seafood consumption varies significantly among individuals, the weighted average activity concentration of ^210^Pb and ^210^Po in seafood samples were adopted for effective ingestion dose evaluation based on the representative values (fish:crustacean:mollusk = 13:1:1) [1]. The total effective ingestion doses ranged from 82.8 to 255 μSv/a for all the age groups. The total effective ingestion dose decreased in the following order: children > juveniles > adults. Among the radionuclides studied, ^210^Po was the highest contributor and accounted for >85% of the total dose. The total effective ingestion dose was also found below the average natural ingestion radiation dose received by humans around the world (300 μSv/a) [1], and the total effective ingestion dose for adults was 1/30 of the public annual effective dose (2.4 mSv/a) caused by natural radiation sources according to the UNSCEAR report [1]. Furthermore the effective dose caused by ^210^Pb and ^210^Po was below the legal dose limit of 1 mSv per year for members of the public recommended by Centre for Environment Fisheries and Aquaculture Science (CEFAS) [36]. Therefore, seafood from the Fujian coast of China is considered to be safe for human consumption.

The lifetime risk of cancer levels associated with the ingestion of ^210^Pb and ^210^Po in seafood were estimated, as shown in Table 4. The risks varied in the range of 6.04 × 10^−6^ (adults, for ^210^Pb) to 1.24 × 10^−4^ (juveniles, for ^210^Po). All the total lifetime risk of cancer from ingestion of ^210^Pb and ^210^Po observed in this study was below the world mean value of 5.3 × 10^−3^ [27], and also much lower the lifetime risk of all cancer (27.77%) in China estimated by [37]. From this point of view, the lifetime risk of cancer due to ^210^Pb and ^210^Po was acceptable.

## 4. Conclusions

The activity concentrations of ^210^Po and ^210^Pb were evaluated in seafood samples collected near the Fujian coast of China. The ^210^Po and ^210^Pb activity concentrations in edible portions of the marine biota were similar to those in the majority of the world’s countries, with the exceptions of Turkey, Spain, and India. ^210^Po was mainly concentrated in the liver, gonad, and intestine samples, and ^210^Pb was mainly concentrated in the bone, fish scale, and head samples. The ^210^Po and ^210^Pb activity concentrations and ^210^Po/^210^Pb ratios observed in this study are valuable references for evaluating the radiation risk of marine biota. In the next research, the relationship between higher ^210^Po activity concentrations and biomarkers (such as H_2_O_2_, Superoxide dismutase and Malondialdehyde) would be also discussed in order to explore the ^210^Po-enriched mechanism in internal organs further. The annual effective ingestion dose and lifetime risk of cancer to humans due to ^210^Po and ^210^Pb for seafood consumption from the Fujian coast were consistent with previous studies performed around the world and were lower than the global mean values. Therefore, the risk of ^210^Po and ^210^Pb in the edible portions of seafood from the Fujian coast of China were within the acceptable ranges to public health.

## Figures and Tables

**Table 1:** Sampling sites, corresponding samples, and feeding habitats.

Sampling Site	Sample	Amount	Feeding Habitat
S1	Yellow croaker (*Larimichthys polyactis*)	18	Artificial rearing area (small fish and shrimp)
S1	Carassius auratus auratus	10	Artificial rearing area (small fish and shrimp)
S1	Red sea bream (*Pagrus major*)	12	Artificial rearing area (small fish and shrimp)
S1	Common Sea perch (*Lateolabrax japonicus*)	11	Artificial rearing area (small fish and shrimp)
S1	Ssargassum	several	Wild
S2	Eel (*Anguillidae*)	several	Wild
S3	Shrimp	several	Wild
S4	Largehead hairtail (*Trichiurus lepturus*)	12	Market

**Table 2:** Dose conversion factors for ingestion (*D*_F_), the risk coefficients (*R*_coe_), and gastrointestinal absorption fractions (*f*_1_) for ^210^Pb and ^210^Po.

Radionuclide	*f*1 (≥1a)	*D*_F_ (Sv/Bq)			*R*_coe_ (risk/Bq)
		Children	Juvenile	Adult	
Pb	0.4 ^a^	2.20 × 10^−6^	1.90 × 10^−6^	6.90 × 10^−7^	3.18 × 10^−8^
Po	0.5	4.40 × 10^−6^	2.60 × 10^−6^	1.20 × 10^−6^	6.09 × 10^−8^

^a^ adult = 0.2.

**Table 3:** Activity concentrations (Bq/kg, *w.w.*) of ^210^Po and ^210^Pb in seafood samples collected from the coast of Fujian, China.

Category	Sample	^210^Po (Bq/kg, *w.w.*)	Uncertainty (*k* = 2, Bq/kg)	^210^Pb (Bq/kg, *w.w.*)	Uncertainty (*k* = 2, Bq/kg)	^210^Po/^210^Pb Ratio
Fish	Yellow croaker (*Larimichthys polyactis*)	1.39	0.15	<MDL		
	Carassius auratus auratus	1.67	0.17	1.69	0.31	0.99
	Red sea bream (*Pagrus major*)	10.4	1. 2	<MDL		
	Common sea perch (*Lateolabrax japonicus*)	0.99	0.12	<MDL		
	Largehead hairtail (*Trichiurus lepturus*)	2.34	0.22	9.24	0.81	0.25
	Eel (*Anguillidae*)	12.6	1.0	11.7	1.1	1.08
Crustacean	Shrimp	12.3	1.0	4.26	0.54	2.88
Algae	Ssargassum	0.74	0.08	<MDL		

MDL: 0.8 Bq/kg wet weight for ^210^Pb.

**Table 4:** Lifetime risk of cancer levels associated with the direct intake of ^210^Pb and ^210^Po in seafood samples.

Lifetime Risk of Cancer	Children	Juveniles	Adults
^210^Po	9.30 × 10^−5^	1.24 × 10^−4^	1.03 × 10^−4^
^210^Pb	1.09 × 10^−5^	1.45 × 10^−5^	6.04 × 10^−6^

## Data Availability

The data presented in this study are available on request from the corresponding author.
